# Association among Executive Function, Physical Activity, and Weight Status in Youth

**DOI:** 10.3390/medicina55100677

**Published:** 2019-10-08

**Authors:** Vaida Borkertienė, Arvydas Stasiulis, Birutė Zacharienė, Laura Kyguolienė, Rasa Bacevičienė

**Affiliations:** 1 Applied physiology and Rehabilitation Department, Lithuanian Sports University, Sport str.6, Kaunas LT-44221, Lithuania; arvydas.stasiulis@lsu.lt (A.S.); birute.zachariene@lsu.lt (B.Z.); 2 Biomedical Department, Panevežio kolegija/University of Applied Sciences, Laivės a. 23, Panevėžys LT-35200, Lithuania; lauravalonyte@yahoo.com (L.K.); rasa.baceviciene@panko.lt (R.B.)

**Keywords:** youth, overweight, normal weight, executive function

## Abstract

*Background and objectives*: Executive function (EF) is an umbrella term that encompasses the set of higher-order processes. Core EFs are inhibition, interference control, working memory, and cognitive flexibility. The aim of the study was to compare the EF between normal weight (NW) and inactive overweight (OW), NW and sport trained (ST), ST and OW 16–19-year-old youths. In addition, the relationship between EF and peak oxygen uptake (VO2peak) was evaluated. *Materials and Methods*: 10 NW, 14 ST, and 10 OW youths participated in this study. EF was evaluated using the ANAM4 battery. VO2peak was measured during an increasing walking exercise (modified Balke test). *Results*: The NW youths demonstrated better visual tracking and attention (94.28% ± 3.11%/90.23% ± 2.01%), response inhibition (95.65% ± 1.83%/92.48% ± 1.05%), speed of processing, and alternating attention with a motor speed component (95.5% ± 3.51%/89.01% ± 4.09%) than the OW youths (*p* < 0.05). The ST youths demonstrated better visual tracking and attention (96.76% ± 1.85%/90.23% ± 2.01%), response inhibition (97.58% ± 0.94%/92.48% ± 1.05%), speed of processing, and alternating attention with a motor speed component (98.35% ± 1.35%/89.01% ± 4.09%) than the OW youths (*p* < 0.05). The ST youths demonstrated better EF results than NW youths (*p* < 0.05). *Conclusions*: The ST 16–19-year-old youths demonstrated better EF than their OW and NW peers. The NW youths demonstrated better EF than their OW peers. There was a significant correlation between VO2peak and EF indicators in all groups of participants.

## 1. Introduction

Cognition is a general term reflecting different processes such as working memory, memory, attention, pattern recognition, executive function (EF), intelligence, concept formation and reasoning, and academic achievement [1]. The executive system can shape multiple executive and behavioral outcomes like specific academic skills and intelligence quotient (scores and overall school achievement) [2].

It is generally agreed that there are three core aspects of EFs: inhibition, working memory, and cognitive flexibility [3]. From these, higher-order EFs are built, such as reasoning, problem-solving, and planning [4,5]. EF plays a significant role in academic achievement.

Working memory is one of the most central EF skills, which continues to develop throughout later childhood [5].

Overweight (OW) youths demonstrate worse working memory skill than normal weight (NW) youths [5]. Youth obesity continues to be a major focus of public health efforts. Obesity has been linked to structural and functional brain abnormalities, particularly in the frontal lobe [6].

Research [7] shows that OW or obese youths have lower self-esteem and self-control than NW youths and that may have an effect on executive achievement. EFs such as attention/concentration, reaction time, processing speed, working memory, visuospatial skills, motor speed, memory, reasoning, and problem-solving are also very important for learning.

OW youths have significantly lower math and reading test scores than non-OW youths in third grade [7,8]. Increased body weight has an effect on general mental ability [9]. Obese youths have twice the rate of executive dysfunction as the normal population [10]. Obese adolescents show slower executive processing speed while maintaining equivalent performance on executive functioning compared with their healthy weight peers [6].

Brain function and structure change significantly during the toddler and preschool years. However, most studies focus on older or younger youths, so the specific nature of these changes is still unclear. There is increasing evidence of important roles for key executive processes, including attention, memory, and learning, in short-term decision-making about eating. There is parallel evidence that people who are OW or obese tend to perform worse on a variety of executive tasks [11].

According to the World Health Organization [12], physical activity can improve cardiorespiratory, muscular fitness, cardiovascular and metabolic health biomarkers, and bone health. It also has psychological benefits, helping to control symptoms of depression and anxiety. Science has proven the importance of the role of physical activity in all age stages [13].

Evidence about the influence of physical activity on EF is mixed. Some researchers have concluded that physical activity has a neutral to positive effect on EF [14]. Resaland with colleagues [15] also assessed the relation between physical activity and academic achievement. Aadland et al. [16] did not find a relation between academic achievement and physical activity. There is the hypothesis that physical activity may influence physiological changes in the brain, and direct physical activity may develop executive skills [17]. Sibley and Etnier [18] found a positive, but weak, correlation between physical activity and different measures of executive performance. Samuel et al. [19] found a significant rise in memory and attention test scores from immediately after exercise to after recovery. Despite much research in this field, it is still not clear what influence physical activity has on EF and how long the effect stays.

However, there are a number of articles that confirm there is no relation between body mass index and academic achievement [9,20]. Obesity and body mass index are negatively related to executive achievement for boys but not girls [21].

Because of controversial results in this field, we organized the investigation of EF and oxygen uptake parameters in three different groups: healthy physically inactive, sport trained, and inactive overweight youths.

The aim of the study was to compare EF among 16–19-years-old healthy physically inactive (NW), sport trained (ST), and overweight (OW) youths.

## 2. Materials and Methods

### 2.1. Participants

In all, 10 healthy physically inactive, NW, 10 OW, and 14 ST 16–19-year-old males participated in this study. Participants of NW and OW groups did not have any specific physical education, except for physical education lessons in school (2 times/week, 45 min/time), which are obligatory for all healthy pupils in Lithuania. OW status was established using age, height, weight, and ≥20% of adipose tissue. Participants of ST groups were soccer players and basketball players with a training experience of 5.0 ± 1.5 years. All ST group youth were invited from Kaunas soccer and basketball clubs. Participants had soccer or basketball training 3 times/week, for 2 hours. Participants participated in soccer/basketball events, regional championships, and matches. All subjects gave their informed consent for inclusion before they participated in the study. The study was conducted in accordance with the Declaration of Helsinki, and the protocol was approved by the Kaunas Regional Ethics Committee, Nr. BE-2-27, 24 May 2017. Written informed consent was obtained from participants (18–19 years old) and from the parents of the participants if the participants were 16–17 years old. The criteria that eliminated participants from the research were: heart disease, diabetes, epilepsy, and musculoskeletal problems. Subjects’ characteristics are recorded in Table 1.

### 2.2. Measurements

#### 2.2.1. Anthropometry

The height of each participant was measured using a stadiometer to the nearest 0.01 m. Body mass and adipose tissue were measured using body composition analyzer “TBF–300” (Japanese) to the nearest 0.1 kg beam. BMI was calculated from body mass (kg) divided by height squared (m^2^).

#### 2.2.2. Pulmonary Gas Exchange

The pulmonary gas exchange parameters were continuously measured breath-by-breath with a portable telemetric system (Oxycon Mobile, Jaeger, Germany). The flow-volume sensor and the gas analyzer (gas mixtures containing 5% CO_2_ and 16% O_2_ were used) were calibrated using automatic calibration procedures, as provided by Jaeger, before each test session.

#### 2.2.3. Heart Rate (HR)

Heart rate was measured with the “Polar” system. Participants wore a wireless chest strap telemetry system to monitor the HR. The chest strap detects the heart’s electrical signals with a sensor that is attached to the strap and turns the signals into heart rate data.

#### 2.2.4. Increasing Walking Exercise (IWE)

IWE was measured by the modified Balke test [22]. Following a 1-min period of standing gas exchange, subjects began a step transition into a 3-min stage at 3 km/h speed and 0% grade. The progressive protocol continued with a 4-min stage at 6 km/h, and the grade of treadmill was increased to 2%, 4%, 6%, 8%, 10%, and as far as the subject could continue. Subjects were verbally encouraged to give maximal effort during the test until volitional exhaustion was achieved. Oxygen uptake data were collected from subjects during test and in resting. 

#### 2.2.5. Executive Function Evaluation

The automated neuropsychological assessment metrics version 4 (ANAM4) was used to evaluate executive function. The four performance tests were, in the order of administration: 2-choice reaction time test (2CRT), code substitution-learning (CSL), go/no-go test, and the simple reaction time (PRO) (Table 2). The specific tests assess areas or domains of executive functioning, including attention/concentration, reaction time, processing speed, working memory, visuospatial skills, motor speed, memory, reasoning, and problem solving. It took ~15–20 min to complete tests for participants [23]. 

### 2.3. Study Design

Participants started the experimental procedure with least intense exercise for 24 h before testing. Participants had to come to the laboratory twice.

The increasing walking exercise (IWE)–Balke was performed the first time in the laboratory. Participants walked on the treadmill at 6 km/h, every minute the treadmill angle was raised 2 degrees during the high-intensity test. The subject continued walking till exhaustion. The peak oxygen uptake (VO2peak) was determined as the highest VO2 within a 20-s period during the IWE [22]. Participants were grouped together in three groups (NW, ST, and OW) by VO2 peak results, adipose tissue, and physical activity. 

EF was tested on the second time in the laboratory. First, the participants learned to do executive tests (performed two trials) and then the true test was recorded for evaluation. It took approximately 20–30 min to complete the executive tests battery.

### 2.4. Data Analysis

VO2 kinetics during IWE were determined using a biexponential model [24]. 

The peak oxygen uptake (VO2peak) was determined as the highest VO2 within a 20-s period during the IWE.

Maximal heart rate (HR max) was measured during IWE. It is the maximal number of heart beats in 60 s.

Maximal pulmonary ventilation (VE max) was measured during IWE. VE is the total flow exhaled per minute. VE = VT × BF, where VT is the ventilation volume and BF is the breathing rate.

Maximal breathing frequency (BF max) is the highest number of breaths a person takes per minute during IWE.

Respiratory exchange ratio (RER) is the ratio between the amount of carbon dioxide (CO_2_) produced in metabolism and oxygen (O_2_) used.

Executive function tests were determined using percent of correct responses [23]:

^#^((NumCorr/(NumCorr + Numinc + NumLapse))) (1)

where ^#^NumCorr is the number of trials with correct response; Numinc is the number of trials with incorrect response; and NumLapse is the number of trials where no response was made in the allotted time.

### 2.5. Statistical Analysis

Statistical analysis was performed using SPSS version 22.0 (IBM Corp., Armonk, NY, USA). The data were tested for normal distribution using the Kolmogorov–Smirnov test; all data were found to be normally distributed. If significant effects were found, Sidak’s post-hoc adjustment was used. Oxygen uptake parameters and EF results were analyzed using two-way ANOVA for the groups’ comparison. Statistical significance was accepted when *p* < 0.05. Calculations of statistical power (SP, as a percentage) were performed for all indicators based on an alpha level of 0.05, sample size (*n* = 34), standard deviations, and changes in the average level of the data. The SP for a significant effect was >80%. The partial eta squared (*η_p_*^2^) was estimated. 

Pearson correlation analysis was performed to determine the correlation of EF with oxygen uptake parameters and weight status. The closer the Pearson correlation coefficient, r, was to either +1 or −1 depended on whether the relationship was positive or negative, respectively. Strength of association was considered as strong from range 0.5 to 1. A *p*-value of <0.05 was considered statistically significant. All values were expressed as mean ± standard deviation.

## 3. Results

### 3.1. Participants’ Characteristics

Table 1 describes the reference characteristics of the participants. All three experimental groups had the same age and height, and there were no significant differences between the groups (*p* > 0.05) (Table 1). However, body weight for the NW group and ST group were lower compared to the OW group (*p* < 0.001, *η_p_*^2^ = 0.54, SP > 100%, *p* < 0.001, *η_p_*^2^ = 0.61, SP > 100%, respectively), and BMI for the NW group and ST group were lower compared to the OW group (*p* < 0.001, *η_p_*^2^ = 0.83, SP > 100%, *p* < 0.001, *η_p_*^2^ = 0.78, SP > 100%, respectively) (Table 1).

### 3.2. Oxygen Uptake Parameters

Oxygen uptake parameters are recorded in Table 3. The NW group had a lower VO_2max_ and VE_max_ (*p* < 0.05, *η_p_*^2^ = 0.33, SP > 88.6%, *p* < 0.05, *η_p_*^2^ = 0.25, SP > 72.4%, respectively) compared to the ST group, but an equally more frequent BF_max_ (*p* < 0.05, *η_p_*^2^ = 0.41, SP > 96.1%). Other oxygen uptake parameters did not differ significantly between the NW and ST groups.

Table 3 shows that the NW group had a higher VO_2_ peak and RER (*p* < 0.05, *η_p_*^2^ = 0.33, SP > 85.3%, *p* < 0.05, *η_p_*^2^ = 0.24, SP > 66.4%, respectively) compared to the OW group. Other oxygen uptake parameters did not differ significantly between the NW and OW groups (*p* > 0.05) (Table 3).

In the ST group, the oxygen uptake parameters: VO_2max_, VO_2peak_, VE_max_, and RER were significantly better than the OW group (*p* < 0.001, *η_p_*^2^ = 0.62, SP > 100%, *p* < 0.001, *η_p_*^2^ = 0.71, SP > 100%, *p* < 0.05, *η_p_*^2^ = 0.34, SP > 85.8%, *p* < 0.05, *η_p_*^2^ = 0.25, SP > 68.7%, respectively) (Table 3). Other oxygen uptake parameters did not differ significantly between the ST and OW groups (*p* > 0.05) (Table 3).

### 3.3. Executive Function Performance

**Figure 1:**
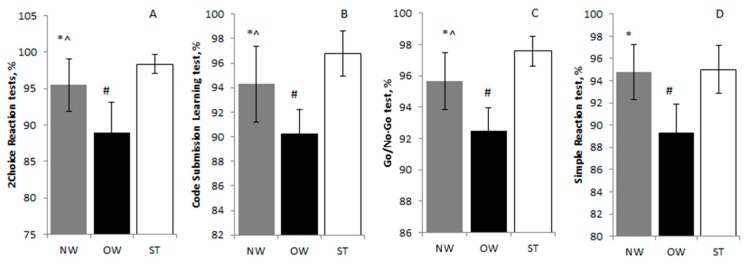
(**A**) 2-choice reaction test, (**B**) code submission learning test, (**C**) go/no-go test, (**D**) simple reaction test results among normal weight (NW), overweight (OW), and sport trained (ST) groups. Values are expressed as means ± standard deviation. * *p* < 0.05 significant difference between NW and OW; ^#^ *p* < 0.05 significant difference between OW and ST; ^ *p* < 0.05 significant difference between NW and ST.

The two choice reaction test (Figure 1A), code submission learning test (Figure 1B), and go/no-go test (Figure 1C) results were significantly different between the NW and OW (95.5 ± 3.51/94.28 ± 3.11/95.65 ± 1.83 and 89.01 ± 4.09/90.23 ± 2.01/92.48 ± 1.05, *p* < 0.05, *η_p_*^2^ = 0.29, SP > 77%; *p* < 0.05, *η_p_*^2^ = 0.19, SP > 55%; *p* < 0.05, *η_p_*^2^ = 0.500, SP > 98%, respectively), OW and ST (89.01 ± 4.09/90.23 ± 2.01/92.48 ± 1.05 and 98.35 ± 1.35/96.76 ± 1.85/97.58 ± 0.94, *p* < 0.05, *η_p_*^2^ = 0.64, SP > 100%; *p* < 0.05, *η_p_*^2^ = 0.65, SP > 100%; *p* < 0.05, *η_p_*^2^ = 0.80, SP > 100%, respectively), and NW and ST (*p* < 0.05, *η_p_*^2^ = 0.02, SP > 63%; *p* < 0.05, *η_p_*^2^ =0.03, SP > 61%; *p* < 0.05, *η_p_*^2^ = 0.01, SP > 72%) youth groups. Simple reaction time test (Figure 1D) results were statistically significant between the NW and OW groups (94.75 ± 2.49 and 89.33 ± 2.56, *p* < 0.05, *η_p_*^2^ = 0.47, SP > 98%, respectively) and ST and OW groups (95.01 ± 2.12 and 89.33 ± 2.56, *p* < 0.05, *η_p_*^2^ = 0.63, SP > 100%, respectively) but not significantly different between NW and ST (*p* > 0.05, *η_p_*^2^ = 0.51, SP > 9.8%) groups.

**Figure 2:**
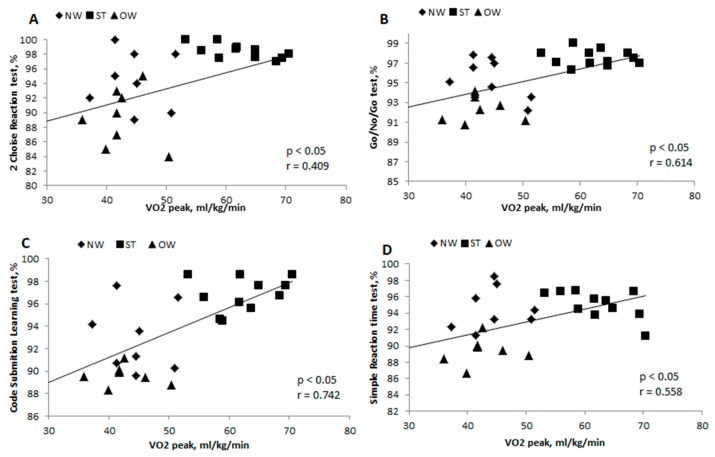
Correlation between 2-choice reaction test and VO2_peak_ (**A**), go/no-go test and VO2_peak_ (**B**), code submission learning test and VO2_peak_ (**C**), simple reaction time and VO2_peak_ (**D**) in normal weight (NW), overweight (OW), and sport trained (ST) groups. r—Pearson correlation coefficient; *p* < 0.05—statistically significant difference.

Correlation between the 2-choice reaction test and VO2peak (Figure 2A) was significantly positive and weak (r = 0.409, *p* < 0.05), go/no-go test and VO2peak (Figure 2B) was significantly positive and strong (r = 0.614; *p* < 0.05), correlation between code submission learning test and VO2 peak (Figure 2C) was significantly positive and strong (r = 0.742; *p* < 0.05), correlation between simple reaction time test and VO2 peak (Figure 2D) was significantly positive and strong (r = 0.558; *p* < 0.05).

## 4. Discussion

The ST 16–19-year-old youths demonstrated better EF than their OW and NW peers. The NW youths demonstrated better EF than their OW peers. Taking all groups together, there was a significant correlation between VO2peak and EF indicators.

Our findings demonstrate that OW youths demonstrate poorer EF than NW physically inactive youths and ST youths. OW youths made more mistakes and spent more time solving executive tests. OW youths’ processing speed and alternating attention with a motor speed component were the worst compared to NW and ST youths. Response inhibition, complex scanning, visual tracking, attention, and visuo-motor response timing were also the lowest of the three groups. Because EF stand out inhibition, shifting, and working memory, these domains are very important for academic achievement. These findings supplement Datar and colleagues [8] findings, that OW youths demonstrate worse academic achievement. Physical activity during class had a positive effect, improving on-task behavior—improving EF [25]. Colcombe and Kramer [26] reported meaningful improvements in EF, processing speed, memory, and motor function. A higher level of physical activity is associated with better neurocognitive performance according to Etnier et al. [27]. On the other hand, Marsh and Kleitman [28] reported negative associations between physical activity and EF. The authors claimed that team sport participants demonstrate poorer cognitive performance than individual sport participants.

Studies investigating EF in OW/obese individuals have claimed that OW/obese demonstrated worse EF [9,29], as well as memory, attention, and motor skills [30] than NW individuals. Li et al. [9] claimed that adiposity may directly negatively influence EF, memory, and learning. Taras et al. [31] concluded obesity had a negative effect on academic performance. Some authors found a negative obesity effect for EF just among girls [32], some just among boys [33]. However, other studies did not find a significant difference in EF among OW/obese and NW individuals [34,35].

The newest research presumes that body mass and EF deficits can be indirectly associated via obesity-induced activation of innate immunity, which directly caused low-grade inflammation in obesity [36,37,38].

NW physically inactive youths showed poorer results in EF tests than ST youths. According to Santana et al. [39], individuals with higher physical fitness execute better EFs such as inhibition, shifting, and working memory. A statistically significant correlation between EF and VO2peak was demonstrated only if all the groups were analyzed together. We found a strong relationship between physical fitness and EF domain in complex scanning, visual tracking and attention, attention and visuo-motor response timing, and response inhibition. The relations between EF domain and choice reaction time and physical fitness were not very strong.

VO2peak reflects endurance capacity and it is the main measurement for assessing aerobic power. OW youths demonstrated lower oxygen uptake peak during the treadmill test than NW and ST youths. Jabbour et al. [40] indicated that the aerobic fitness of inactive obese children was significantly different from those of active obese and non-obese children.

It is known that EF might be improved by computer training [41], repeated practice [42], and physical activity [43]. On the other hand, some researchers did not find a relation between physical activity and EF [44,45,46].

There are mechanisms that might explain the relationship between physical activity and executive functioning. De Bruijn et al. [47] grouped these mechanisms into two groups: physiological mechanisms and learning/developmental mechanisms. Physiological mechanisms claim that different levels of physical activity lead to supplementation of the brain’s plasticity and leads to an increase of neurotransmitters resulting in changes in the brain [1,17,48]. Learning/developmental mechanisms explain the relation between physical activity and EF indicating a learning process while being physically active [18,47]. ST children are characterized as having larger brain volumes in the basal ganglia and hippocampus. This relates to a higher performance on tasks of memory and cognitive control and also to a higher brain function during tasks of cognitive control and better scores on tests of academic achievement [49,50], increasing oxygen saturation and glucose delivery, improving cerebral blood flow, and increasing neurotransmitters levels [3]. Using MRI and measuring the electrical activity of the brain, positive differences in structural brain volumes and brain function were established [49,51]. Wu and coworkers [5] found an early obesity or OW effect and future learning results.

Our findings support the hypothesis that physical activity has a positive influence on EF. We evaluated three different groups with the same cognitive test battery and same protocol for oxygen uptake kinetics. Because cognitive function evaluation, physical activity level, and groups in previous studies were different, we believe our findings give new contribution to this field, and allow us to better understand the importance of physical activity and harm of becoming overweight for youth. We know little about how useful physical activity can be; therefore, longitudinal research is necessary to investigate it more.

## 5. Conclusions

The ST 16–19-year-old youths demonstrated better EF (choice reaction time, complex scanning, visual tracking, attention, response inhibition) than their OW and NW peers. The NW youths demonstrated better EF than their OW peers. Taking all groups together there was a significant correlation between VO2peak and EF indicators.

## Figures and Tables

**Table 1:** Characteristics of participants.

Variable	NW Group (*n* = 10)	ST Group (*n* = 14)	OW Group (*n* = 10)
Age (year)	18.12 ± 0.60	18.66 ± 0.32	18.75 ± 0.50
Height (m)	1.76 ± 0.14	1.79 ± 0.06	1.81 ± 0.03
Weight (kg)	68.65 ± 14.31 *	75.24 ± 6.16 ^#^	89.95 ± 6.75
Body mass index (kg/m^2^)	21.78 ± 1.45 *	23.45 ± 1.80 ^#^	27.45 ± 1.61
Adipose tissue (%)	17 ± 2.81 *	15 ± 1.71 ^#^	24 ± 3.87

NW—normal weight group; ST—sport trained group; OW—overweight group. Values are means ± standard deviation. * *p* < 0.05 when comparing NW group and OW group; ^#^ *p* < 0.05 when comparing ST group and OW group.

**Table 2:** Descriptions of the executive tests [23].

Test	Description of Test	Executive Domain
2CRT	This test measures choice reaction time by presenting the user with a “*” or “o” on the display. The subject is instructed to respond as quickly as possible by pressing the designated button for each stimulus as soon as the stimulus appears.	This test measures choice reaction time.
CSL	In this test, the subject must compare a displayed digit-symbol pair with a set of defined digit-symbol pairs or the key. The user presses designated buttons to indicate whether the pair in question represents a correct or incorrect mapping relative to the key. In the learning phase, the defined pairs are presented on the screen along with the digit-symbol pair in question.	Results of this test are used as an index of complex scanning, visual tracking, and attention.
Go/No-Go	The subject is presented with two characters, “x” and “o”. The subject is instructed to respond as quickly as possible to the “x” by pressing a button each time the stimulus appears. When the “o” appears, the user is to do nothing.	This test assesses response inhibition.
PRO	The subject clicks the left mouse button (single-button response) when an asterisk stimulus is presented on the screen. This stimulus is presented at different intervals for 40 trials, and the reaction time for each trial is recorded. This subtest assesses reaction time.	Results of this test are used as an index of attention and visuo-motor response timing.

2CRT—2-Choice Reaction Time test; CSL—Code Substitution-Learning, PRO—Simple Reaction Time.

**Table 3:** Oxygen uptake parameters.

	NW Group	ST Group	OW Group
VO_2_max, L/ min	2.89 ± 0.52 ^	4.83 ± 0.53 ^#^	3.86 ± 0.65
VO_2_peak, mL/kg/min	43.62 ± 6.15 *	64.19 ± 6.45 ^#^	42.97 ± 5.49
HR_max_, b/min	194 ± 8.4	191 ± 9.3	190 ± 8.2
VE_max_, L/min	101.9 ± 17.01 ^	123 ± 15.55 ^#^	103.3 ± 9.73
VT_max_, L	3.03 ± 1.19	3.5 ± 0.32	3.20 ± 0.28
BF_max_, L/min	42.54 ± 4.23 ^	40.33± 5.91	43.09 ± 5.72
RER	1.19 ± 0.10 *	1.18 ± 0.12 ^#^	1.11 ± 0.04
Max test power, W	317.47 ± 11.96	353.93 ± 5.90	309.50 ± 13.59

HR—heart rate, VE—minute ventilation, BF—breathing frequency, VT—ventilation volume, RER—respiratory exchange ratio; max test power—maximal workload during the test, b—beat in a minute; NW—normal weight group; ST—sport trained group; OW—overweight group. Values are means ± standard deviation. ^ *p* < 0.05 when comparing NW group and ST group; * *p* < 0.05 when comparing NW group and OW group; ^#^ *p* < 0.05 when comparing ST group and OW group.
